# Functional Magnetic Resonance Imaging in the Evaluation of the Elastic Properties of Ascending Aortic Aneurysm

**DOI:** 10.21470/1678-9741-2018-0406

**Published:** 2019

**Authors:** Kaushal Kishore Tiwari, Stefano Bevilacqua, Giovanni Donato Aquaro, Pierluigi Festa, Lamia Ait-Ali, Tommaso Gasbarri, Marco Solinas, Mattia Glauber

**Affiliations:** 1 Department of Cardiothoracic and Vascular Surgery, College of Medical Sciences, Teaching Hospital, Bharatpur, Chitwan, Nepal.; 2 Fondazione Toscana Gabriele Monasterio (FTGM), G. Pasquinucci Heart Hospital, Department of Adult Cardiac Surgery, Via Aurelia Sud, Massa, Italy.; 3 Istituto di Scienze della Vita, Scuola Superiore Sant’ Anna, Piazza Martiri della Libertа, Pisa, Italy.; 4 Fondazione Toscana Gabriele Monasterio (FTGM), MRI Laboratory, CNR, Via Moruzzi, Pisa, Italy.; 5 Instituto Clinico Sant’Ambrogio, Research Hospital, Gruppo San Donato, Milan, Italy.

**Keywords:** Aortic Aneurysm, Thoracic, Systole, Diastole, Aorta, Elastic, Dilatation, Pathology, Magnetic Resonance Imaging

## Abstract

**Objective:**

To evaluate the aortic wall elasticity using the maximal rate of systolic distension (MRSD) and maximal rate of diastolic recoil (MRDR) and their correlation with the aortic size index (ASI).

**Methods:**

Forty-eight patients with thoracic aortic aneurysm were enrolled in this study. A standard magnetic resonance imaging (MRI) protocol was used to calculate MRSD and MRDR. Both MRSD and MRDR were expressed as percentile of maximal area/10^-3^ sec. ASI (maximal aortic diameter/body surface area) was calculated. A correlation between MRSD, MRDR, ASI, and the patient’s age was performed using regression plot.

**Results:**

A significant correlation between MRSD (t=-4,36; r^2^=0.29; *P*≤0.0001), MRDR (t=3.92; r^2^=0.25; *P*=0.0003), and ASI (25±4.33 mm/m^2^; range 15,48-35,14 mm/m^2^) is observed. As ASI increases, aortic MRSD and MRDR decrease. Such inverse correlation between MRSD, MRDR, and ASI indicates increased stiffness of the ascending aorta. A significant correlation between the patient’s age and the decrease in MRSD and MRDR is observed.

**Conclusion:**

MRSD and MRDR are significantly correlated with ASI and the patient’s age. They seem to describe properly the increasing stiffness of aortas. These two new indexes provide a promising, accessible, and reproducible approach to evaluate the biomechanical property of the aorta.

**Table:** 

Abbreviations, acronyms & symbols				
ASI	= Aortic size index		MRDR	= Maximal rate of diastolic recoil
ATAA	= Ascending thoracic aortic aneurysm		MRSD	= Maximal rate of systolic distension
BAV	= Bicuspid aortic valve		NEX	= Number of excitations
BSA	= Body surface area		SPSS	= Statistical Package for the Social Sciences
ECG	= Electrocardiogram		SSFP	= Steady-state free-precession
E_inc_	= Incremental elastic modulus		STJ	= Sinotubular junction
FVP	= Flow velocity propagation		TAV	= Tricuspid aortic valve
IRAD	= International Registry of Aortic Dissection		TE	= Echo time
MRI	= Magnetic resonance imaging		TR	= Repetition time

## INTRODUCTION

Ascending aorta aneurysm is a virulent, lethal, but indolent disease, having a mortality rate of about 90% in case of acute complications^[1]^. Current guidelines suggest prophylactic surgery at a maximal diameter of 5.5 cm in otherwise healthy patients, while 4-5 cm in patients with Marfan and other congenital syndromes^[2,3]^. However, these criteria have several limitations. Data from the International Registry of Aortic Dissection (IRAD) have shown that most of the patients operated on for acute type A aortic dissection had a diameter < 5.5 cm, and 40% of them with diameter < 5 cm^[4]^. In this version of IRAD report, Pape et al. stressed that “methods other than size measurement of ascending aorta are needed to identify patients at risk for dissection”. Davies et al. have suggested the aortic size index (ASI) (maximal aortic diameter/body surface area) as a predictor of aortic aneurysm rupture or dissection in order to better define the timing of surgery^[5]^. Recently, two new magnetic resonance imaging (MRI) indexes of aortic wall elastic properties, named maximal rate of systolic distension (MRSD) and maximal rate of diastolic recoil (MRDR), have been proposed to assess the velocity of aortic wall distension and recoil during the cardiac cycle^[6]^. The aim of our study is to evaluate the aortic wall elasticity, using these two new indexes, in adult patients with aneurysm or ectasia of thoracic aorta and their correlation with ASI.

## METHODS

Altogether, forty-eight consecutive patients were enrolled in this study. In detail, twenty-seven patients out of 125 patients operated on for aortic root and/or ascending aorta ± other procedure were enrolled in this study. Furthermore, twenty-one prospective patients, undergoing clinical and instrumental follow-up for ascending aortic and/or root ectasia were included. All patients underwent a functional MRI. The following exclusion criteria were used: patients with Marfan syndrome, familiar aortic aneurysm, Ehler-Danlos syndrome, aortic dissection, and patients with contraindication to MRI or refusing the enrollment. This study was approved by the local ethical committee and a written consent was taken from every participating patient.

### MRI Protocol

All patients underwent comprehensive cardiac MRI study using 1,5 T Signa CV/i MRI scanner (GE, Milwaukee, Wisconsin, USA) with an 8-channel cardiac phase array coil. A standard protocol to acquire the MRI images was followed as proposed by Aquaro et al.^[6]^.

Briefly, the thoracic aorta was visualized by acquiring sagittal-oblique cine images, parallel to the major aortic axis, using a breath-hold, electrocardiogram (ECG)-triggered, steady-state free-precession (SSFP) pulse sequence with the following parameters: 400-mm field of view, 8-mm slice thickness, no gap, 1 number of excitations (NEX), 12 views per segment, echo time (TE)/repetition time (TR) 1.6/3.2, flip angle 45º, matrix 224 x 224, and reconstruction matrix 256 x 256. The number of cardiac phases was set according to the heart rate to obtain an aortic wall excursion temporal resolution of approximately 10^-3^ sec. Cross-sectional cine SSFP images with the same parameters were acquired at different aortic levels: 1) at the aortic valve plane to evaluate the aortic valve morphology and to quantify the planimetric area; 2) at the aortic root (measurement of aortic root diameter was performed in para-sagittal SSFP slice obtaining a plane that includes right coronary Valsalva sinus and left coronary Valsalva sinus); 3) at the sinotubular junction (STJ); 4) at the proximal ascending aorta (5 mm above the STJ) to measure MRSD and MRDR; and 5) at the level of maximum diameter of the ascending aorta.

**Fig. 1:**
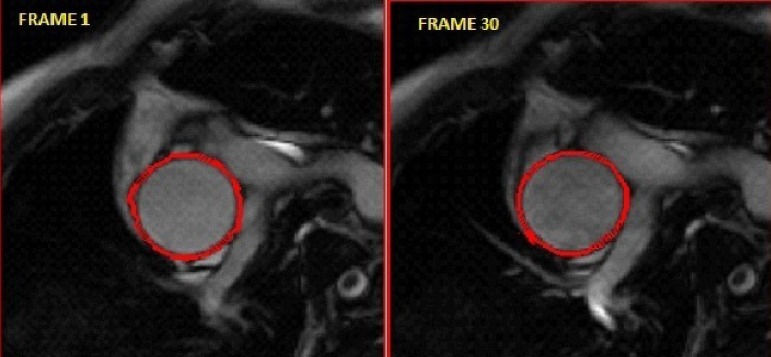
Manual processing of acquired magnetic resonance images showing first steady-state free-precession frame and the frame with the maximal cross-sectional area of the ascending aorta.

**Fig. 2:**
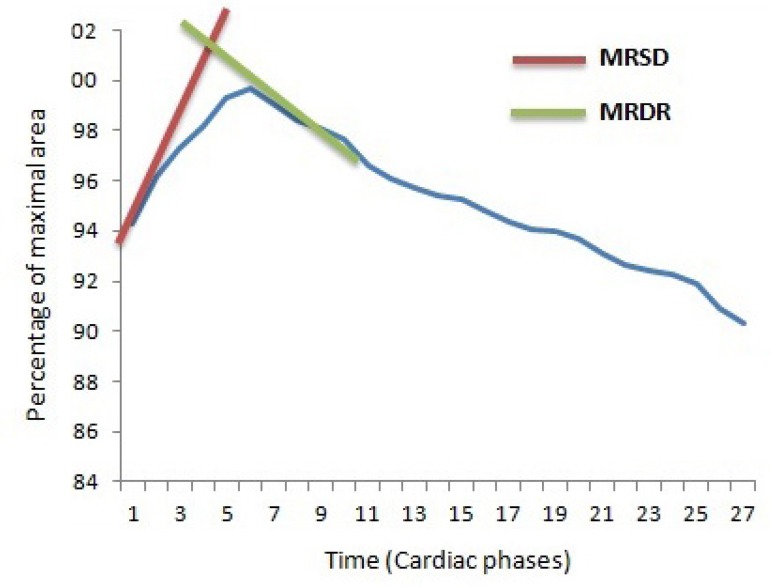
Area/time curve of the ascending aorta. MRDR=maximal rate of diastolic recoil; MRSD=maximal rate of systolic distension.

A manual processing of all images was done using a dedicated computer program, developed in our institution as described by Aquaro et al.^[6]^ (Figure 1). Consequently, MRSD and MRDR were calculated as cross-sectional area of the proximal ascending aorta (5 mm above the STJ) measured in each cardiac phase. MRSD and MRDR were measured 5 mm over the STJ as previously described by Aquaro et al.^[6]^. The rationale of this choice was to obtain a homogeneously round-shaped cross section of ascending aorta (then excluding the aortic root) in order to simplify the measurement and to contemporary standardize the acquisition of SSFP images for all the patients. These parameters were indexed for the maximal end-systolic cross-sectional area and plotted against the time (relative cross-sectional area/time curve, Figure 2). In this curve, MRSD was measured as the maximal systolic upslope and MRDR was measured as the maximal diastolic down slope. MRSD and MRDR were expressed as percentile of maximal area/10^-3^ sec.

Body surface area of all the patients was calculated using the Mosteller formula. ASI was calculated for all patients using the formula: ascending aortic maximum diameter divided by body surface area^[5]^. Ascending aortic diameter was measured using the same MRI images in all patients.

### Statistical Analysis

Categorical variables were compared between groups by Fisher’s exact test when appropriate. For the comparison of continuous variables, *t*-tests were performed when the variable distribution was found to be normal by the Kolmogorov-Smirnov test; otherwise, a nonparametric Mann-Whitney U test was used. Group data were summarized by mean and standard deviation or by frequency percentages. The relationships between variables and ASI were evaluated by single and multiple linear regression analysis. Statistical significance was considered for a probability value < 0.05. Statistical elaboration was conducted using the Statistical Package for the Social Sciences (SPSS) software (SAS Institute, SPSS Inc, Chicago, IL), version 14.

## RESULTS

**Fig. 3:**
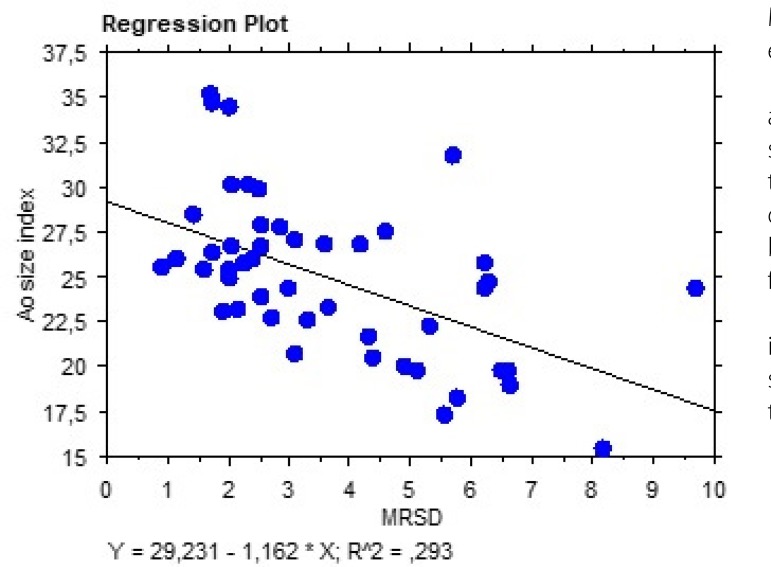
Regression plot showing a significant correlation between maximal rate of systolic distension (MRSD) and aortic size index.

**Fig. 4:**
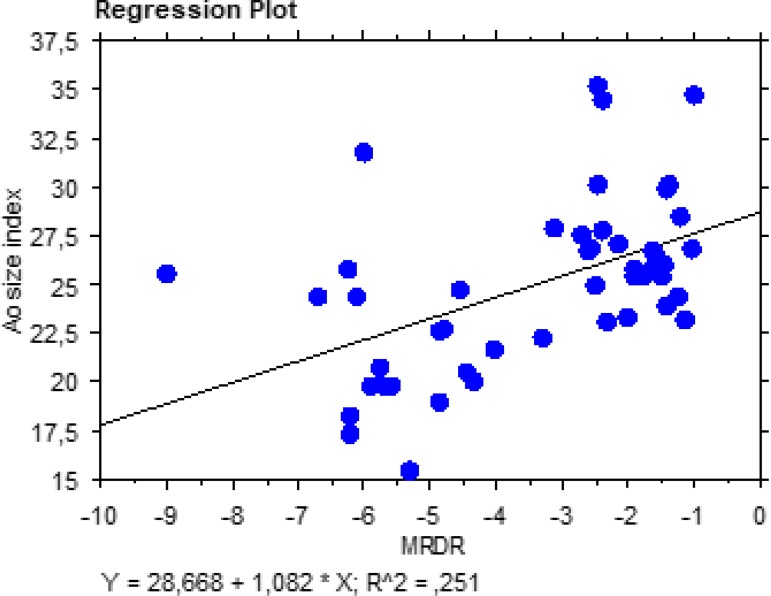
Regression plot showing a significant correlation between maximal rate of diastolic recoil (MRDR) and aortic size index.

**Fig. 5:**
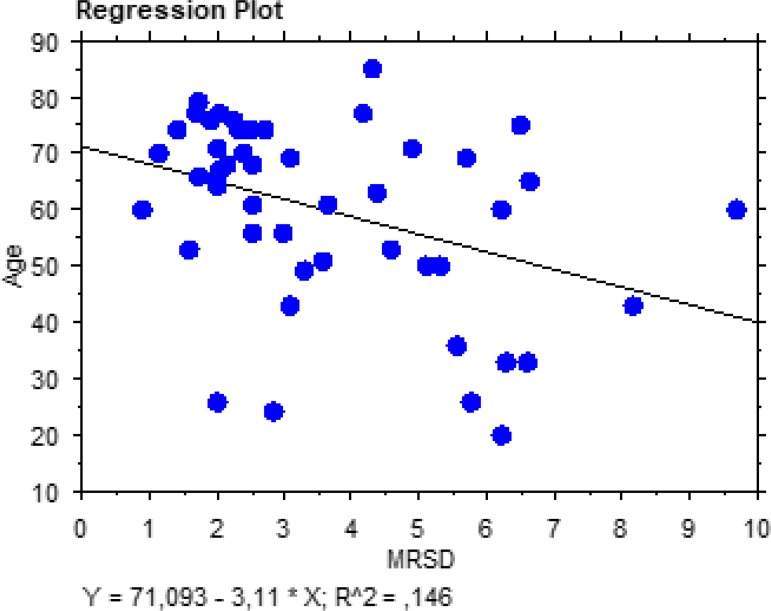
Regression plot showing a significant correlation between maximal rate of systolic distension (MRSD) and the patients’ age.

**Fig. 6:**
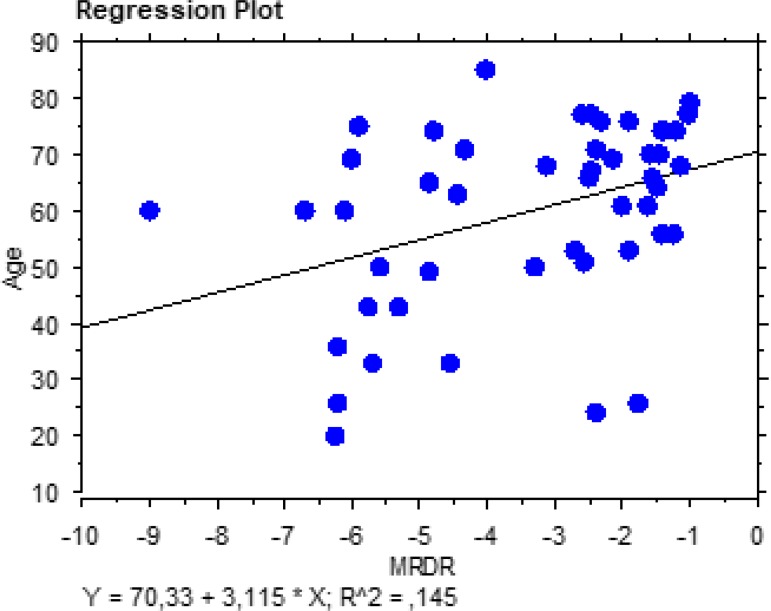
Regression plot showing a significant correlation between maximal rate of diastolic recoil (MRDR) and the patients’ age.

Demographic and clinical characteristics of patients are described in Table 1. Mean aortic root and ascending aortic diameter were 46.58±6.65 mm and 42.77±5.72 mm, respectively. Most patients, *i.e*., 33 patients (68.2%) had predominant aortic insufficiency. Out of the total 48 patients, 23 (47.9%) patients had grade 3+ aortic regurgitation and nine (18.75%) patients had grade 4+ regurgitation. Fifteen patients with predominantly aortic stenosis had mean gradient of 43±16.98 mmHg. Nineteen patients (39.58%) had bicuspid aortic valve (BAV). ASI was 25.00±4.33 mm/m^2^ (range 15.48-35.13 mm/m^2^). Mean ASI for the 21 non-operated patients was 22.58±4.01 cm/m^2^. Out of 27 patients who underwent surgeries, 15 had aortic valve replacement with ascending aorta replacement and 12 underwent aortic root replacement (nine, Bentall procedure; three, Tirone David procedure). Ten patients from this group were operated on by ministernotomy, two patients by minithoracotomy, and the remaining 15 patients by conventional full sternotomy approach. Mean MRSD and MRDR were 3.64±2.01 and -3.39±2.00, respectively. As shown in Figures 3 and 4, there was a significant simple correlation between MRSD (t=-4.36; r^2^=0.29; *P*≤0.0001), MRDR (t=3.92; r^2^=0.25; *P*=0.0003), and ASI. As ASI increases, MRSD and MRDR decrease (MRDR becomes less negative), meaning that wall stiffness is positively related to aortic dilation. Furthermore, we observed a significant correlation between the patients’ age and these two MRI indexes (MRSD: t=-2.80; r^2^=0.15; *P*=0.0073; MRDR: t=2.79; r^2^=0.15; *P*=0.0076), confirming, once again, a stiffening of the aortic wall with progressive ageing (Figures 5 and 6). Moreover, on multivariate analysis, the only factor that maintained a significant relationship with ASI was MRSD (t=-2.073; *P*=0.0440). These indexes were not related to the type of predominant aortic valve disease (aortic stenosis *vs*. regurgitation). Additionally, when we analyzed MRSD and MRDR values among two different groups of patients with tricuspid aortic valve (TAV) and BAV, we didn’t find any significant differences in these subgroups of patients (MRSD for TAV 3.49±1.64 *vs*. BAV 3.92±2.51, *P*=0.43; MRDR for TAV -3.12±1.68 *vs*. BAV -3.79±2.4, *P*=0.26).

**Table 1:** Characteristics of the 48 patients enrolled in this study.

N^o^	Characteristics	Values
1	Male	37 patients (77.1%)
2	Female	11 patients (22.9%)
3	Age	59.77±16.39 years
4	BSA	1.88±0.18 m^2^
5	Aortic size index	25.00±4.33 cm/m^2^
6	Aortic root diameter	42.77±5.72 mm
7	Ascending aortic diameter	46.58±6.65 mm
8	Bicuspid aortic valve	19 patients (39.58%)
9	Aortic insufficiency	33 patients (68.7%)
10	MRSD	3.64±2.01
11	MRDR	-3.38±2.00
12	Maximal aortic area	20.16±5.56 mm^2^
13	Minimal aortic area	17.64±5.22 mm^2^
14	Dyslipidemia	9 patients (18.75%)
15	Smoking	22 patients (45.83%)
16	Hypertension	24 patients (50%)
17	Diabetes	3 patients (6.25%)
18	Operated	27 patients (56.25%)

BSA=body surface area; MRDR=maximal rate of diastolic recoil; MRSD=maximal rate of systolic distension

## DISCUSSION

The present study highlights the potential role of these new MRI indexes, firstly described by Aquaro et al.^[6]^ in a group of young BAV patients, in ascending thoracic aortic aneurysm (ATAA) adult patients affected by aneurysm or ectasia of ascending aorta and/or aortic root^[6]^. MRSD and MRDR are expressions of the systolic and diastolic aortic strain rate. Basically, these two indexes explain the biomechanics of the aortic wall during the systolic and diastolic phases of the cardiac cycle. Indeed, slow MRSD and MRDR velocities in aneurysm patients could reveal the impaired elasticity of aortic wall.

Rupture of the aorta occurs when the stress exerted on the aortic wall exceeds the aortic wall capacity to sustain it^[7,8]^. A stiff aorta is prone to rupture due to its inability to cope with the pressure wave. Davies et al.^[5]^ classified the risk of rupture/dissection according to the ASI^[5]^. Therefore, the correlation between MRSD, MRDR, and ASI suggests a potential role of functional MRI in individualizing patients at risk.

Aortic diameter is still the main criteria for surgical indication in clinical practices. Nevertheless, if we see data from different studies, including IRAD, absolute aortic size itself is insufficient to predict if an aorta dissects or ruptures. Furthermore, there are several surgical centers, including ours, that are using ASI as an indication for surgery, in addition to the absolute size. Davies et al.^[5]^ have reported a strong association of aortic rupture and aortic dissection with increasing ASI (*P*=0.0022 and 0.0215, respectively). Additionally, they have found out that when rupture and dissection are considered together, ASI remains predictive of negative events. So, we emphasize that ASI is a reliable tool for predicting morbid conditions and a significant correlation between MRSD, MRDR, and ASI could describe the impaired elastic property of the aorta vulnerable to acute complication.

Until now, elastic property of the aorta has been evaluated mostly in *ex vivo* condition using aortic wall specimens derived from the operated patient^[8-11]^, but an *in vivo* evaluation of the elastic property by echocardiography or MRI gives clinicians an extra clue to decide the timing for intervention. Other characteristics of the aorta, like distensibility, aortic stress, and incremental elastic modulus (E_inc_), have been evaluated using preoperative and intraoperative echocardiography^[12,13]^. In a study, we have observed that MRI can be used to evaluate accurate diameter, distensibility, and flow wave velocity of the aorta^[14]^. In these studies, it has been shown that large aneurysms (>5cm) were stiffer (lower distensibility) and experienced higher wall stress and higher E_inc_ values than small aneurysms. However, a large variation in aortic distensibility was previously reported; this could be partially explained by the fact that distensibility is influenced by aortic dimensions and by systemic pressure. Additionally, because aortic biophysical properties change with increasing pressure due to the recruitment of collagen fibers, distensibility reflects only the mean of aortic elastic behavior in the physiologic pressure range^[15]^. Flow velocity propagation (FVP) using MRI has been anticipated as a useful tool for evaluating the elastic properties of ascending aortic wall^[16]^. Both BAV patients and Marfan patients had significantly faster FVP than controls^[6]^. The reduced aortic wall elasticity in BAV patients could be the cause of the increased flow velocity in the thoracic aorta. However, FVP could be influenced by the hemodynamic status of the patients, potentially increasing under hyperdynamic conditions and decreasing in patients with left ventricular dysfunction or aortic disease.

The new indexes, MRSD and MRDR, are both independent of central aortic pressure as demonstrated previously by Donato Aquaro et al.^[6]^. Therefore, they seem to be more accurate in the assessment of the elastic property of the aorta. MRSD and MRDR are measured using relative changes (in % of maximum) of aortic cross-sectional area over time. The advantage of this “relative” and not “absolute” evaluation of aortic area changes as well as of the choice of evaluating the maximal rates of these changes is that the measurement of this index is not conditioned by the presence of aortic dilation and by the value of pulse pressure. Evaluating the rate of distension, MRSD allows the evaluation of kinetic/potential energy. Kinetic energy produced by ventricular contraction is partially absorbed by the aortic wall and transferred to potential energy by the elastic distension. Then, in diastole, that potential energy is again transformed in kinetic energy, allowing the diastolic flow. The rate of systolic distension is directly related to the energy absorption. Then we hypothesized that MRSD may be related to ventricular stroke work more than blood pressure. However, further studies are needed to verify this hypothesis.

Furthermore, we performed multivariate analysis, in which the only factor that maintains a significant relationship with ASI was MRSD. This finding highlights predominantly systolic distension property impairment of the aortic wall in aneurysmal patients. Such correlation with ASI, which is the risk predictor of aortic rupture and dissection, further implies a noncompliance aorta during the systolic stress. In fact, aortic dissection during emotional stress, sexual activity, strenuous physical activity, and heavy weight training causing rise in systolic blood pressure to over 300 mmHg could be result of decreased MRSD in this patients^[17,18]^.

Donato Aquaro et al.^[6]^ didn’t report any difference in MRSD and MRDR between BAV patients with or without aortic dilation, suggesting also an independence of aortic diameter. However, this apparent contradiction to our results could be explained by the larger aortic diameters (aortic diameter 44.95±6.6 mm; ASI 23.55±3.65 mm/m^2^) and older age (50.05±15.22 years) of our BAV patients.

Several studies^[9,19,20]^ have shown the effect of age on the major reduction of ascending aorta elasticity. Age-related deterioration of the mechanical property of aorta is due to accumulated fatigue and age-related changes in wall composition. In a recent study using magnetic resonance, Aquaro et al.^[21]^ have shown MRSD and distensibility decreasing progressively through the classes of age after an initial plateau between 20 and 30 years in males and 15 and 20 years in females. In our study, a significant correlation between MRSD, MRDR, and the patients’ age shows the efficacy of these new indexes in measuring and predicting the elastic property of the aorta *in vivo*. Furthermore, MRSD can be used as a predictor for progression of aortic aneurysm, identifying patients with fast progression of ascending aorta dilatation^[22]^.

Iliopoulos et al.^[8]^ have concluded that ATAA development is not associated with mechanical weakening but with the stiffening and reduction in the vessel extensibility^[8]^. Furthermore, Vorp et al.^[11]^ have explained aortic rupture as a result of increased wall stress caused by tissue stiffening and vessel enlargement. These two arguments together make sense of the relevance of aortic stiffness *in vivo* evaluation to predict the likelihood of aortic dissections or rupture in ATAA patients.

### Limitation

Limitations of this study include a small number of study population and no healthy matched control group due to the difficulty in enrollment of healthy matched control subjects. Nevertheless, to compensate this limitation we have analyzed the relationship between ASI, MRSD and MRDR, which is considered an index of severity of the disease. By using this approach, we have surpassed the arbitrary distinction between aneurysmal and non-aneurysmal patients. Considering these limitations, any conclusion should be interpreted with caution. Nonetheless, it could be helpful in future to perform a multicenter study enrolling a large number of patients.

Another point to reveal is that we haven’t performed histological analysis of the aortic specimens from the operated patients, however we think it would be another interesting study to evaluate these patients’ aortic specimens, which will provide a complete insight into the pathophysiological aspect of this lethal pathology.

## CONCLUSION

This significant correlation between MRSD, MRDR, ASI, and age could make them promising, accurate, and potential indexes to evaluate the elastic property of the aorta *in vivo*. Such accessible and reproducible approach to evaluate the biomechanical property of the aorta, beyond the sole measurement of the maximal diameter, could allow us to select, in a better way, patients at risk of acute complications, leading to a more appropriate timing for surgical treatment.

**Table:** 

Author's roles & responsibilities	
KKT	Substantial contributions to the conception or design of the work; or the acquisition, analysis, or interpretation of data for the work; drafting the work or revising it critically for important intellectual content; final approval of the version to be published
SB	Substantial contributions to the conception or design of the work; or the acquisition, analysis, or interpretation of data for the work; drafting the work or revising it critically for important intellectual content; agreement to be accountable for all aspects of the work in ensuring that questions related to the accuracy or integrity of any part of the work are appropriately investigated and resolved; final approval of the version to be published
GDA	Substantial contributions to the conception or design of the work; drafting the work or revising it critically for important intellectual content; agreement to be accountable for all aspects of the work in ensuring that questions related to the accuracy or integrity of any part of the work are appropriately investigated and resolved; final approval of the version to be published
PF	The acquisition, analysis, or interpretation of data for the work; agreement to be accountable for all aspects of the work in ensuring that questions related to the accuracy or integrity of any part of the work are appropriately investigated and resolved; final approval of the version to be published
LAA	The acquisition, analysis, or interpretation of data for the work; agreement to be accountable for all aspects of the work in ensuring that questions related to the accuracy or integrity of any part of the work are appropriately investigated and resolved; final approval of the version to be published
TG	Final approval of the version to be published
MS	The acquisition, analysis, or interpretation of data for the work; agreement to be accountable for all aspects of the work in ensuring that questions related to the accuracy or integrity of any part of the work are appropriately investigated and resolved; final approval of the version to be published
MG	Substantial contributions to the conception or design of the work; drafting the work or revising it critically for important intellectual content; agreement to be accountable for all aspects of the work in ensuring that questions related to the accuracy or integrity of any part of the work are appropriately investigated and resolved; final approval of the version to be published
